# Multifaceted roles of S100A6 in neurological disorders: expression, interaction networks, and clinical implications

**DOI:** 10.3389/fphar.2026.1890559

**Published:** 2026-08-12

**Authors:** Jing Zhang, Peng Tang, Linshu Huang, Jianghao Lv, Ying Chen, Yun Wang, Yi Luo

**Affiliations:** 1 Department of Laboratory Medicine, Zhongnan Hospital of Wuhan University, Wuhan, Hubei, China; 2 Hubei Provincial Clinical Research Center for Molecular Diagnostics, Wuhan, Hubei, China; 3 Department of Anesthesiology, Zhongnan Hospital of Wuhan University, Wuhan, Hubei, China; 4 Hubei Clinical Research Center for Emergency and Resuscitation, Zhongnan Hospital of Wuhan University, Wuhan, Hubei, China

**Keywords:** biomarker, calcium signaling, glial cells, glioma, neurodegenerative diseases, S100A6

## Abstract

S100A6 (calcyclin) is a multifunctional Ca^2+^/Zn^2+^-binding protein of the S100 family, widely expressed in neurons and glia with a developmentally regulated and cell-type-specific pattern. This review synthesizes current knowledge on its roles in the nervous system. We detail its complex interactome, which includes cytoskeletal regulators, molecular chaperones, nuclear transport proteins, and cell surface receptors, positioning S100A6 as a central signaling hub. Furthermore, we examine its dynamic and context-dependent involvement in major neurological disorders. In Alzheimer’s disease and amyotrophic lateral sclerosis, it functions as a glial-derived factor linking protein aggregation, metal dyshomeostasis, and neuroinflammation. In neuro-oncology, S100A6 exhibits dual roles, acting as a promoter of malignancy and immunosuppression in glioblastoma, an epigenetically silenced marker in medulloblastoma, and a diagnostic aid for peripheral nerve sheath tumors. Its expression is also altered in epilepsy, traumatic brain injury, and autoimmune encephalitis. Understanding the nuanced functions of S100A6 offers significant potential for developing novel diagnostic biomarkers and targeted therapeutic strategies for a range of challenging neurological conditions.

## 1 Introduction

S100A6, also termed calcyclin, is a member of the S100 family of calcium-binding proteins characterized by their distinctive EF-hand structural motifs. Encoded by a gene located on human chromosome 1q21, the 90-amino-acid protein S100A6 monomer contains two EF-hand domains that confer its ability to bind Ca^2+^ with differential affinity: a lower-affinity atypical site at the N-terminus and a higher-affinity canonical site at the C-terminus (M et al., 2004; Calabretta et al., 1986; Otterbein et al., 2002). Upon Ca^2+^ binding, S100A6 undergoes a significant conformational rearrangement, exposing hydrophobic surfaces critical for target protein recognition and facilitating its role in intracellular Ca^2+^ signaling cascades (Sastry et al., 1998). Beyond calcium, S100A6 also exhibits Zn^2+^ binding capacity through a distinct molecular site, thereby expanding its functional repertoire as a divalent cation sensor and potential modulator of zinc-mediated processes (Filipek et al., 1990).

S100A6 was initially characterized by its strong association with cell proliferation and motility. These properties, which have since been extensively linked to tumorigenesis, invasiveness, and metastasis across various cancers, underscore its role in oncogenic processes. Beyond this, S100A6 is now recognized as a pleiotropic regulator that influences a broad spectrum of fundamental cellular activity (Chen et al., 2024; Wang et al., 2021a; Wang et al., 2017). These include cytoskeletal remodeling, cell cycle progression, differentiation programs, calcium homeostasis maintenance, and the regulation of apoptotic pathways (Słomnicki and Leśniak, 2010; Leclerc et al., 2007). Within the complex architecture of the nervous system, S100A6 demonstrates intricate and developmentally regulated spatiotemporal expression patterns across diverse neuronal and glial populations (Tiu et al., 2000; Yamashita et al., 1999; Bartkowska et al., 2017; Hagmeyer et al., 2019; Yamada and Jinno, 2014; Kjell et al., 2020; Yamashita et al., 1997; Yamada and Jinno, 2012). Its expression is dynamically modulated in response to physiological stimuli, cellular stress, injury, and within the pathological milieu of numerous neurological disorders, including Alzheimer’s disease (AD), amyotrophic lateral sclerosis (ALS), epilepsy, traumatic brain injury (TBI), and various neuro-oncological conditions (Leclerc et al., 2007; Bartkowska et al., 2017; Jurewicz et al., 2013; Fang et al., 2014).

Despite accumulating evidence that positions S100A6 as a significant player in both neural physiology and disease pathogenesis, the precise molecular mechanisms governing its actions within the central and peripheral nervous systems remain incompletely defined. Critical knowledge gaps persist regarding its cell-type and context-specific functions, the detailed signaling networks it engages, its secretory mechanisms and extracellular functions, and the stoichiometry of its interactions with a vast array of binding partners under physiological versus pathological conditions. This comprehensive review aims to synthesize the current knowledge on S100A6, focusing on its expression profile, protein-protein interaction network, and multifaceted roles across a spectrum of neurological disorders. This analysis seeks to assess the emerging potential of S100A6 as a key pathological mediator and a candidate biomarker or therapeutic target in neurological diseases.

## 2 Stage- and cell-type-specific expression of S100A6 in the nervous system

Within the intricate framework of the nervous system, S100A6 expression follows a sophisticated regulatory program, exhibiting distinct stage-specific patterns that govern its distribution across cellular compartments and brain regions. This expression is markedly characterized by pronounced regional and cell-type specificity throughout different life stages.

Under normal physiological conditions, the expression profile of S100A6 differs notably between the human fetal and aged cerebral cortex, suggesting a dynamic functional repertoire. Detailed studies indicate that S100A6 protein expression displays clear spatiotemporal specificity in key regions of the fetal brain, including the hippocampus, entorhinal cortex, and occipital cortex. A particularly intriguing pattern is observed in the mid-gestational hippocampus, where its immunoreactivity intensifies within specific subsets of neurons and glial cells before subsequently diminishing (Tiu et al., 2000). This transient peak implies distinct, stage-specific roles for S100A6 in the developmental programming of different brain areas.

From a cellular perspective, S100A6 demonstrates a broad yet selective distribution. Early immunohistochemical studies in rodent models confirmed its expression in a wide array of neuronal types, such as pyramidal neurons in the hippocampus and cortex, cerebellar granule cells, brainstem neurons, and olfactory receptor neurons. Concurrently, it is present in multiple glial cell lineages, including astrocytes in white matter tracts, certain ependymal cells, and Schwann cells in the peripheral nervous system (Yamashita et al., 1999; Bartkowska et al., 2017; Hagmeyer et al., 2019). Higher-resolution investigations using multiplex immunofluorescence have further refined this map, revealing that S100A6 is highly expressed in neural stem cells co-labeled with markers such as sex determining region Y-box2 (Sox2), brain lipid-binding protein (BLBP), and glial fibrillary acidic protein (GFAP). In the adult mouse hippocampal dentate gyrus, a critical neurogenic niche, S100A6 is particularly abundant in astrocytic precursors (Yamada and Jinno, 2014; Kjell et al., 2020). Functional evidence underscores the importance of this expression, as inhibition of S100A6 specifically curtails astrocyte generation without affecting neurogenesis, and its sustained expression proves crucial for the final maturation of astrocytes.

**FIGURE 1:**
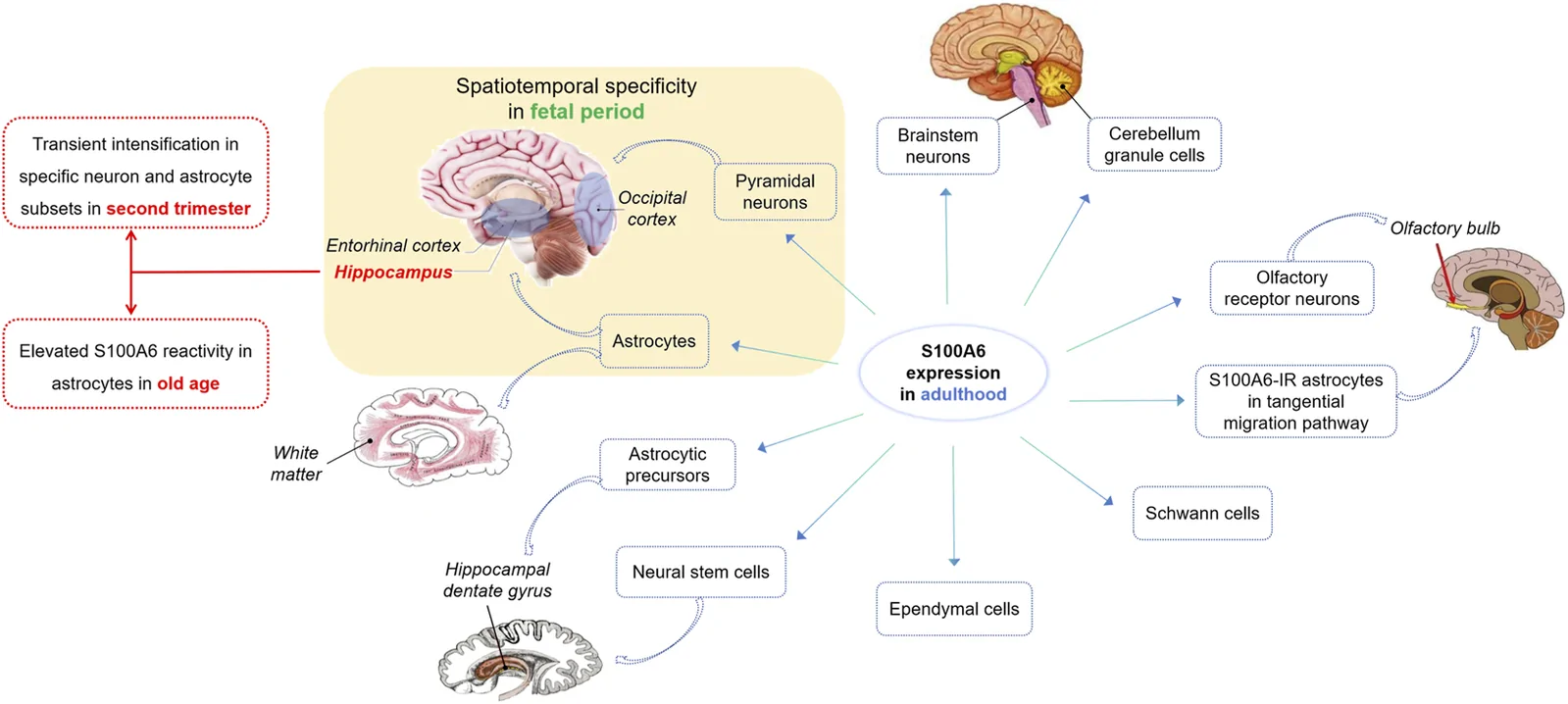
Lifespan-dependent, stage- and cell-type-specific expression of S100A6 in the nervous system. The schematic highlights the well-characterized expression landscape in adulthood, where S100A6 is broadly expressed in neurons (pyramidal, cerebellar granule, brainstem, and olfactory receptor neurons), glia (astrocytes, ependymal cells, and Schwann cells), and neural stem/astrocytic precursors in the hippocampal dentate gyrus. Limited data from other stages indicate transient fetal spatiotemporal specificity (second-trimester peak in hippocampal neurons/astrocytes) and elevated reactivity in aged hippocampal astrocytes.

Furthermore, S100A6 expression correlates with specific neurobiological processes. For instance, within the tangential migratory pathway guiding neuroblasts to the olfactory bulb in adult rodents, S100A6 is precisely localized to a subpopulation of astrocytes. This strategic positioning suggests a potential collaborative role with these glial cells in facilitating neuronal migration and circuit integration (Yamashita et al., 1997). S100A6 expression is also subject to modulation by age and physiological state, as evidenced by increased S100A6 reactivity observed in hippocampal astrocytes of aged mice (Yamada and Jinno, 2012). Collectively, these intricate, stage- and cell-type-specific expression patterns form the foundational molecular landscape through which S100A6 participates in nervous system development, plasticity, and homeostasis (Figure 1). This complex regulation also provides the underlying basis for its multifaceted and often dichotomous functions in various pathological conditions, ranging from its potential roles in repair and protection to its involvement in promoting disease progression.

## 3 Interaction networks and functional partners of S100A6 in the brain

The diverse biological functions of S100A6 are executed through its dynamic interactions with a wide spectrum of intracellular and extracellular partners. We have categorized the S100A6 interactome into five functional modules based on localization and downstream pathway convergence, collectively enabling S100A6 to act as a central signaling hub (Table 1).

**TABLE 1:** Functional modules of the S100A6 interactome in the nervous system.

Module	Representative interactors	Interaction mode	Function	Disease relevance
Calcium signaling modulators	- CacyBP/SIP (Filipek and Wojda, 1996; Jastrzebska et al., 2000; Filipek et al., 2002; Matsuzawa and Reed, 2001; Filipek et al., 2008; Kilanczyk et al., 2009; Wasik et al., 2016) - Sgt1 (Kitagawa et al., 1999; Spiechowicz and Filipek, 2005; Nowotny et al., 2003; Prus and Filipek, 2011)	- Ca^2+^-dependent binding - Compete with other partners	- Microtubule dynamics - Cell growth - Cell differentiation - Chaperone complex assembly	- Neurodegeneration (Wasik et al., 2013)
Chaperone/Co-chaperone regulators	Hop, Cyp40, FKBP52, CHIP, PP5 (Shimamoto et al., 2008; Shimamoto et al., 2014; Yamaguchi et al., 2014; Yamaguchi et al., 2012; Shimamoto et al., 2010; Shimamoto et al., 2013; Sakane et al., 2017)	TPR domain-mediated binding	- Protein folding and degradation - Mitochondrial import - Phospho-signaling	- Proteinopathies (Haldar et al., 2020; Rossi et al., 2025; Botelho et al., 2012) - Cellular stress responses (Shimamoto et al., 2010; Shimamoto et al., 2013; Sakane et al., 2017)
Cytoskeletal organizers	Actin, tropomyosin, tubulin, TPPP, annexins (Jurewicz et al., 2020; Golitsina et al., 1996; Filipek et al., 1996; Robaszkiewicz et al., 2021; Wills et al., 1994; Khan et al., 2019; Simon et al., 2020; Rezvanpour and Shaw, 2009; Doi et al., 2021)	- Direct binding - Ternary complex formation	- Microfilament and microtubule dynamics - Centrosome/cilia function - Membrane-cytoskeleton linkage	- Glioma invasion (Kucharczak et al., 2001) - Injury repair (Fang et al., 2014; Ding et al., 2020)
Extracellular mediators	- RAGE (Donato et al., 2017; Yatime et al., 2016) - Integrin β1 (Donato et al., 2017; Jurewicz et al., 2018)	Receptor-ligand binding	- Inflammatory signaling - Cell adhesion - DAMP activity	- Neuroinflammation (Donato et al., 2017; Yatime et al., 2016) - Autoimmune encephalitis (Tsai et al., 2019; Lin et al., 2023)
Nuclear mediators	- p53 (Słomnicki et al., 2009; van Dieck et al., 2010; Króliczak et al., 2008; Wang et al., 2023) - Importin α (Takata et al., 2010) - Lamin A/C (Kilańczyk et al., 2012)	- Ca^2+^-dependent binding - Inhibit NLS recognition	- Transcriptional regulation - Nucleocytoplasmic transport - Nuclear envelope integrity	- Apoptosis dysregulation (Słomnicki et al., 2009; van Dieck et al., 2010) - Cell cycle checkpoint defects (Króliczak et al., 2008; Wang et al., 2023)

CacyBP/SIP, calcyclin-binding protein/Siah-1-interacting protein; TPR, tetratricopeptide repeat; PP5, protein phosphatase 5; TPPP, tubulin polymerization-promoting protein; RAGE, advanced glycation end-products; DAMP, damage-associated molecular pattern; NLS, nuclear localization signals.

### 3.1 Intracellular signaling and regulatory partners

S100A6 engages with key intracellular proteins that link calcium signaling to cellular regulation. A principal partner is the calcyclin-binding protein/Siah-1-interacting protein (CacyBP/SIP), which is abundantly expressed in the brain (Filipek and Wojda, 1996; Jastrzebska et al., 2000; Filipek et al., 2002; Matsuzawa and Reed, 2001). This Ca^2+^-dependent interaction sequesters CacyBP/SIP, modulating its availability to bind other partners such as tubulin and extracellular signal-regulated kinases 1/2 (ERK1/2), thereby influencing microtubule dynamics and cell growth and survival pathways (Filipek et al., 2008; Kilanczyk et al., 2009). S100A6 binding also regulates the phosphorylation state of CacyBP/SIP (Wasik et al., 2016). Another crucial ligand is Sgt1, involved in cell cycle regulation and chaperone complex assembly (Kitagawa et al., 1999). Sgt1 is broadly expressed in rat tissues, with highest levels in brain, particularly in cerebellum and cortex (Spiechowicz and Filipek, 2005). Despite limited sequence similarity with CacyBP/SIP, Sgt1 binds S100A6 and other S100 proteins in a Ca^2+^-dependent manner (Nowotny et al., 2003). This binding promotes nuclear translocation of Sgt1 during stress and controls its chaperone interactions, thereby regulating cellular stress responses. Additionally, S100A6 binding prevents phosphorylation of its binding site on Sgt1, a modification that does not affect the interaction itself (Nowotny et al., 2003; Prus and Filipek, 2011).

Beyond these specific ligands, S100A6 interacts with a suite of cytoplasmic proteins containing tetratricopeptide repeat (TPR) domains (Shimamoto et al., 2008; Shimamoto et al., 2014; Yamaguchi et al., 2014; Yamaguchi et al., 2012). This group comprises molecular chaperones and co-chaperones (Hop, Cyp40, FKBP52, CHIP, FKBP38), motor protein components (kinesin light chain), the mitochondrial import receptor Tom70, protein phosphatase 5 (PP5), and centrosomal proteins such as FOR20 and FOP (Shimamoto et al., 2010; Shimamoto et al., 2013; Sakane et al., 2017). These interactions position S100A6 at the nexus of critical cellular processes, including protein folding and degradation, intracellular transport, mitochondrial function, and phospho-signaling (Supplementary Figure S1).

### 3.2 Cytoskeletal interactors and structural regulation

A major facet of S100A6 function is its direct regulation of cytoskeletal architecture. It binds directly to both globular (G-actin) and filamentous actin (F-actin), as well as specific tropomyosin isoforms (e.g., Tpm1.8), forming ternary complexes that modulate microfilament organization and stability (Jurewicz et al., 2020). Its influence extends to actin-binding proteins such as myosin, caldesmon, cofilin-1, calponin, and non-muscle myosin IIA, thereby regulating actomyosin contractility (Golitsina et al., 1996; Filipek et al., 1996; Robaszkiewicz et al., 2021; Wills et al., 1994; Khan et al., 2019; Simon et al., 2020). Through interactions with various annexin (II, VI, XI), S100A6 also impacts membrane-cytoskeleton linkages (Rezvanpour and Shaw, 2009).

Concurrently, S100A6 engages with the microtubule network via α/β-tubulin heterodimers and tubulin polymerization-promoting protein (TPPP), as well as centrosomal proteins such as OFD1, regulating centrosome function and ciliogenesis (Sakane et al., 2017; Doi et al., 2021). This dual engagement with both microfilament and microtubule systems underscores its role as a coordinator of integrated cytoskeletal remodeling affecting cell shape, adhesion, and motility (Supplementary Figure S1).

### 3.3 Extracellular engagement and nuclear targets

S100A6 can be secreted and functions as a damage-associated molecular pattern (DAMP) (Anfinogenova et al., 2020; Supplementary Figure S2). Its primary cell surface receptor is the receptor for advanced glycation end-products (RAGE). Binding to the C1 domain of RAGE triggers pro-inflammatory signaling cascades, including reactive oxygen species (ROS) generation and c-Jun N-terminal kinase (JNK) activation, impacting proliferation, differentiation, and apoptosis (Donato et al., 2017; Yatime et al., 2016). S100A6 also interacts in a Ca^2+^-dependent manner with integrin β1, activating downstream kinases (ILK, FAK, PAK) to regulate cell adhesion and motility (Donato et al., 2017; Jurewicz et al., 2018).

Within the nucleus, S100A6 interacts with proteins governing nuclear structure and transport (Supplementary Figure S3). It binds nuclear lamin A/C, suggesting a potential role in nuclear envelope integrity (Kilańczyk et al., 2012). Notably, S100A6 binds importin α, inhibiting the import of proteins with classical nuclear localization signals (NLS), thereby proposing a novel mechanism for regulating nucleocytoplasmic transport during calcium transients (Takata et al., 2010). Most significantly, S100A6 interacts with the tumor suppressor p53 and its family members p63/p73 in a Ca^2+^-dependent manner (Słomnicki et al., 2009; van Dieck et al., 2010). This interaction enhances the transcriptional activity of p53, increasing expression of pro-apoptotic target genes and sensitizing cells to oxidative stress-induced death. This establishes a direct molecular link between S100A6-mediated calcium signaling and the p53 pathway, governing critical decisions in cell cycle arrest and apoptosis (Króliczak et al., 2008; Wang et al., 2023).

## 4 Pathological manifestations of S100A6 in neurological disorders

Like other S100 family members, S100A6 regulates various nervous system pathologies and its expression changes following neural injury (Yang et al., 2023). Although the pathological functions of S100A6 are diverse, they can be reconciled within a unifying framework defined by three interdependent contextual determinants. First, compartmentalization dictates functional outcome. Intracellular S100A6 modulates calcium signaling, cytoskeletal dynamics, and transcription via cytoplasmic and nuclear partners, whereas extracellular S100A6 engages surface receptors such as RAGE to initiate inflammatory cascades. Second, the responding cell type critically shapes the response. In neurons, S100A6 interacts with CacyBP/SIP and p53 to influence apoptosis, whereas in astrocytes its effects range from cytoprotection to neurotoxicity depending on disease stage and microenvironment. Third, the local interactome composition varies across cell types and pathological states, determining signaling directionality through differential engagement of partners including CacyBP/SIP, Sgt1, and p53. These determinants frequently co-operate, explaining the context-specific roles of S100A6 across neurological disorders. The following sections examine S100A6 expression patterns and functional roles in neurodegenerative diseases, brain tumors and other neurological disorders (Table 2).

**TABLE 2:** Expression patterns and functional roles of S100A6 in neurological disorders.

Diseases	Expression	Mechanisms	Potential application
Alzheimer’s disease (AD)	Enriched in Aβ plaque-associated reactive astrocytes (Boom et al., 2004; Hagmeyer et al., 2019)	Initial: Zinc chelation may destabilize Aβ plaques (Tian et al., 2019) Progression: Drives tau hyperphosphorylation via CacyBP/SIP (Wasik et al., 2013); Enhances PPP5C-mediated tau dephosphorylation (Haldar et al., 2020); RAGE binding exacerbates neuroinflammation and neuronal loss (Ast et al., 2022)	Candidate biomarker and therapeutic target for neuroinflammatory pathways
Amyotrophic Lateral Sclerosis (ALS)	Upregulated in activated astrocytes near degenerating motor neurons (Hoyaux et al., 2000; Hoyaux et al., 2002; Iridoy et al., 2018)	- Disrupts astrocytic glutamate uptake, promoting neuronal excitotoxicity (Yamada and Jinno, 2012) - As a Ca^2+^/Zn^2+^ sensor linked to zinc dyshomeostasis (Rossi et al., 2025) - Binds and accelerates mutant SOD1 aggregation (Botelho et al., 2012)	Therapeutic target for modulating glia-driven pathology
Glioma/Glioblastoma	Increased expression in glioma does not correlate with tumor grade due to heterogeneity (Camby et al., 1999; Camby et al., 2000) S100A6 expression can be induced by factors like gastrin in glioblastoma (Kucharczak et al., 2001)	- Promotes tumor cell invasion and motility (Kucharczak et al., 2001) - High expression correlates with an immunosuppressive tumor microenvironment (Hong et al., 2023; Han et al., 2025)	Prognostic biomarker indicating poorer outcome
Medulloblastoma	Silenced S100A6 expression by promoter hypermethylation (Lindsey et al., 2007; Rehman et al., 2005)	Promoter hypermethylation associated with aggressive large cell/anaplastic histological variant (Lindsey et al., 2007)	Epigenetic biomarker for diagnosis and prognosis
Neurothekeomas	Histologically high sensitivity and strong immunoreactivity in cellular neurothekeoma (Plaza et al., 2009; Fullen et al., 2003)	—	Histopathological classification and differential diagnosis of peripheral nerve sheath tumors
Epilepsy	Persistently upregulated in patients and animal models (Jurewicz et al., 2013; Lee et al., 2007; Cacheaux et al., 2009)	S100A6 upregulation closely linked to neuronal injury and epileptogenesis (Jurewicz et al., 2013; Kobori et al., 2002)	Marker for epileptic seizures and neuronal damage
Traumatic brain injury (TBI)	Biphasic expression pattern (Fang et al., 2014; Ding et al., 2020)	Initial decrease: correlates with acute impairment Later recovery: aligns with functional improvement and neural repair (Fang et al., 2014; Ding et al., 2020)	Marker for brain injury repair process
Postoperative neurocognitive dysfunction (po-NCD)	Increased levels in neurons of hippocampus and prefrontal cortex (Li et al., 2021)	Associated with pathophysiology for learning and memory (Li et al., 2021)	Tissue-based biomarker for po-NCD.
Autoimmune encephalitis (AE)	Elevated S100A6 expression and its promoter hypomethylation (Lin et al., 2023)	Disrupts BBB integrity to promote B-cell infiltration, contributing to disease initiation (Tsai et al., 2019)	Contributor and target in AE-related autoimmune dysregulation

Aβ, β-amyloid; CacyBP/SIP, calcyclin-binding protein/siah-1-interacting protein; PPP5C, protein phosphatase 5, catalytic subunit; RAGE, receptor for advanced glycation end-products; SOD1, superoxide dismutase 1; BBB, blood-brain barrier.

### 4.1 Neurodegenerative diseases

Neurodegenerative diseases, characterized by the progressive loss of specific neuronal populations alongside sustained neuroinflammation, present a complex pathogenic landscape where cell-autonomous and non-cell-autonomous mechanisms intertwine. The dysregulated expression of S100A6 in these conditions positions it as a significant molecular player, with its roles demonstrating both shared themes and disease-specific nuances (Filipek and Leśniak, 2020).

In Alzheimer’s disease (AD), the most prevalent form of dementia, S100A6 exhibits a striking and specific upregulation within reactive astrocytes, most pronounced in those forming clusters in close physical association with β-amyloid (Aβ) plaques in both gray and white matter (Boom et al., 2004; Hagmeyer et al., 2019; De Bastiani et al., 2023). The functional implications of this elevated expression are complex and stage-dependent. During initial phases, S100A6 may participate in compensatory or protective responses. *In vitro* evidence suggests its zinc-chelating property can solubilize aggregated Aβ, potentially mitigating plaque stability (Tian et al., 2019). As AD progresses and chronic inflammation ensues, sustained high levels of astrocytic S100A6 are thought to contribute to pathogenesis through multiple intersecting mechanisms (Cristóvão and Gomes, 2019). At the mechanistic level, two calcium-dependent pathways have been experimentally validated: (i) S100A6 binding to CacyBP/SIP inhibits CacyBP/SIP-mediated tau dephosphorylation, indirectly promoting hyperphosphorylated tau accumulation (Wasik et al., 2013); (ii) S100A6 enhances PPP5C phosphatase activity specifically toward phosphorylated tau (Haldar et al., 2020). Beyond protein aggregation, secreted S100A6 may engage RAGE to modulate neuroinflammatory signaling and disrupt synaptic maintenance pathways, although this extracellular axis remains primarily inferred from receptor-binding studies rather than direct *in vivo* evidence (Ast et al., 2022). Consistent with these findings, transcriptome meta-analyses have identified S100A6 as positively correlated with the AD phenotype, positioning it as a candidate genetic marker (Wruck et al., 2016). Collectively, the current evidence establishes S100A6 as a glial factor mechanistically linked to both Aβ and tau pathologies *in vitro*. However, the translation of these pathways to human AD pathogenesis, as well as the relative contribution of S100A6 compared with other glial-derived factors, awaits validation in longitudinal patient cohorts and cell-type-specific *in vivo* models.

In Amyotrophic lateral sclerosis (ALS) patient tissue and models, S100A6 is robustly upregulated in activated astrocytes residing in affected motor regions, observed in both sporadic and familial (e.g., SOD1-mutant) forms (Hoyaux et al., 2000; Hoyaux et al., 2002; Iridoy et al., 2018). The upregulation of this Ca^2+^/Zn^2+^-binding protein may be linked to disrupted zinc homeostasis, a process particularly relevant given the metal-binding properties of mutant SOD1 (Rossi et al., 2025). Two mechanistic links have been established *in vitro*: (i) S100A6 physically interacts with and accelerates aggregation of mutant SOD1 (Botelho et al., 2012); (ii) Astrocytic S100A6 contributes to glutamate dysregulation, potentially via effects on astrocytic glutamate transporters, thereby exacerbating excitotoxicity (Yamada and Jinno, 2012). What remains unresolved is whether S100A6 upregulation represents a primary pathogenic driver or a secondary response to neuronal injury, as no conditional knockout studies have been reported in ALS models. This distinction is critical for evaluating S100A6 as a therapeutic target, since targeting a secondary amplifier may have different efficacy and safety profiles than targeting a primary driver.

In summary, across AD and ALS, S100A6 operates as a critical glial-derived factor whose dysregulation is closely linked to core disease processes, including protein misfolding and aggregation, disruption of metal ion homeostasis, and dysfunctional neuron-glia communication. Its actions can span a spectrum from initially adaptive to ultimately maladaptive. This highlights the dual nature of glial responses in neurodegeneration and establishes S100A6 as a potential focal point for therapeutic strategies aimed at modulating neuroinflammatory and neurotoxic pathways.

### 4.2 Brain tumors

S100A6 exhibits a complex and context-dependent role in oncogenesis, functioning as a modulator of key cancer hallmarks including uncontrolled proliferation, evasion of apoptosis, and metastatic dissemination. Its impact is mediated through diverse signaling pathways, such as p38/MAPK and PI3K/Akt, and its expression often correlates with tumor cell aggressiveness (Yang et al., 2023; Leśniak and Filipek, 2023). However, its function is not uniformly pro-tumorigenic, as evidenced by its anti-proliferative effect in certain lung cancer cell lines, highlighting its cell-type-specific nature (Wang et al., 2021b). This variable expression profile and functional duality position S100A6 as a molecule of significant interest in neuro-oncology, particularly in tumors of glial and neural origin.

In glioma, the clinical and pathological significance of S100A6 has been investigated with seemingly divergent findings. Early studies suggested potential utility in histological grading, showing higher protein levels in high-grade versus low-grade astrocytomas (Camby et al., 1999). However, subsequent work confirmed S100A6 upregulation in gliomas relative to normal brain but found no consistent correlation with pathological grade or proliferation index, indicating significant intra-tumoral heterogeneity (Camby et al., 2000). More recent mechanistic evidence indicates that S100A6 can be upregulated in glioblastoma by factors such as gastrin, enhancing tumor cell motility and invasiveness (Kucharczak et al., 2001). Beyond these observations, high S100A6 expression in glioblastoma is associated with an immunosuppressive tumor microenvironment, characterized by increased infiltration of regulatory T cells and myeloid-derived suppressor cells alongside reduced effector T cells, and correlates strongly with poorer prognosis (Hong et al., 2023; Zhang et al., 2021). These associations are robust across multiple transcriptomic datasets, including spatial and single-cell transcriptomics analyses, yet they remain correlative (Yang et al., 2024; Han et al., 2025). Whether S100A6 directly drives immunosuppression or merely marks a broader mesenchymal program remains experimentally unresolved. This distinction carries practical implications. If S100A6 is a functional driver, it may serve as a therapeutic target, whereas if it is only a correlated bystander, efforts should shift to the upstream regulators that coordinate the immunosuppressive phenotype.

Medulloblastoma exemplifies the epigenetic regulation of S100A6. Promoter hypermethylation silences S100A6 expression in a subset of these tumors, and this hypermethylation is clinically associated with the aggressive large cell/anaplastic variant and worse prognosis (Lindsey et al., 2007; Rehman et al., 2005). By contrast, the hypomethylated and overexpressed S100A4 in other samples illustrates the heterogeneous epigenetic landscape of S100 genes in this malignancy (Lindsey et al., 2007; Tang et al., 2020). For neurothekeomas, a distinct category of benign peripheral nerve sheath tumors, S100A6 serves a well-established diagnostic role, particularly in the histologically challenging cellular variant (Misago et al., 2004; Plaza et al., 2009). Its high sensitivity and strong immunoreactivity, especially when combined with loss of pan-S100 or negative for keratin, provides diagnostic value in distinguishing these tumors from melanoma mimics (Plaza et al., 2009; Fullen et al., 2003).

### 4.3 Other neurological disorders

Beyond neurodegeneration and brain tumors, S100A6 expression is dynamically altered in several other neurological conditions, although the evidence base varies considerably. In epilepsy, S100A6 is consistently upregulated in both patients and animal models, showing a close association with seizure activity and neuronal injury (Jurewicz et al., 2013; Lee et al., 2007; Cacheaux et al., 2009). Its elevation in key limbic structures following induced status epilepticus, along with high spatiotemporal correlation with markers of neuronal damage, further solidifies its link to the epileptogenic process (Jurewicz et al., 2013; Kobori et al., 2002). By contrast, traumatic brain injury (TBI) exhibits a distinct biphasic expression pattern. An initial decrease in hippocampal levels coincides with acute damage, whereas a subsequent recovery-phase increase parallels functional improvement and expression of neural repair markers (Fang et al., 2014; Ding et al., 2020). In postoperative neurocognitive dysfunction (po-NCD), elevated S100A6 protein in learning- and memory-related brain regions has been observed in affected patients, suggesting a tissue-based biomarker candidate (Li et al., 2021). A more functionally characterized role emerges in autoimmune encephalitis (AE), where S100A6 promotes B lymphocyte migration across the blood-brain barrier, likely through altered endothelial permeability, and its promoter hypomethylation in patient cells may underlie its elevated expression (Tsai et al., 2019; Lin et al., 2023). Collectively, these associations establish S100A6 as a broadly reactive marker of neurological pathology, but with the notable exception of autoimmune encephalitis, direct mechanistic involvement remains largely inferred from correlative patterns rather than functional validation.

## 5 Future perspectives and conclusion

Despite significant advances in understanding the roles of S100A6 in the nervous system, numerous questions remain to be explored. First, mechanistic insights into cell-type and context-specific functions are urgently needed. While S100A6 is expressed in both neurons and glia, its precise actions likely differ based on cellular origin, disease stage, and microenvironment. Employing cell-type-specific knockout models, for example, in astrocytes versus neurons, combined with spatial transcriptomics and proteomics will be crucial to dissect these context-dependent roles (Stuart and Satija, 2019; Saunders et al., 2018). Understanding how S100A6 signaling diverges, such as promoting survival in one context while exacerbating damage in another, is key to harnessing its therapeutic potential. Second, the secretory mechanisms and extracellular functions of S100A6 warrant deeper investigation. The pathways governing its release from neural cells, potentially via exosomes or unconventional secretion, and the concentration gradients it forms in the extracellular space during injury or disease are poorly defined (Basso et al., 2016; Chauhan et al., 2022). Maybe, its interactions with components of the extracellular matrix and other DAMPs could modulate its bioavailability and function, influencing neuroinflammatory cascades in conditions like AE and TBI.

Moreover, translating S100A6 into a clinically useful biomarker requires rigorous validation. Its presence in biofluids such as cerebrospinal fluid (CSF) or blood, potentially as part of a multi-analyte panel, should be explored in large, longitudinal patient cohorts for diseases including AD, ALS, and malignant gliomas (Hong et al., 2023; Teunissen et al., 2022). However, several limitations that must be addressed before clinical application. First, assay standardization remains lacking, with reported cut-off values varying considerably across studies. Second, S100A6 elevation is observed across diverse neurological insults, compromising its specificity for any single condition (Yang et al., 2023; Filipek and Leśniak, 2020). By contrast, established biomarkers such as neurofilament light chain (NfL) have demonstrated higher specificity for axonal injury and have been recognized as a surrogate endpoint supporting regulatory approval in *SOD1*-ALS (Wiesenfarth et al., 2024). S100B, though widely studied as a biomarker of neural distress, similarly suffers from limited specificity in broader neurological contexts (Michetti et al., 2023). Third, prospective validation in independent cohorts and head-to-head comparisons with established benchmarks are absent for S100A6. These gaps do not diminish its potential value but underscore that it remains a candidate marker requiring further scrutiny. Standardizing detection assays and establishing clear cut-off values linked to disease stage, progression, or treatment response will be essential steps. Finally, exploring S100A6 as a therapeutic target is a promising yet challenging frontier. Strategies could involve small molecules or biologics designed to disrupt specific pathogenic interactions, such as S100A6-RAGE in neuroinflammation or S100A6-integrin in metastasis, or to modulate its expression (Leclerc et al., 2007; Donato et al., 2017). Given its dual neuroprotective and neurotoxic roles, any therapeutic intervention must be highly targeted to avoid disrupting its essential physiological functions. Epigenetic modulators to reverse its silencing in tumors such as medulloblastoma, or approaches to correct its dysregulated expression in reactive astrocytes, represent intriguing possibilities (Zhang et al., 2013; Qureshi and Mehler, 2018).

In conclusion, S100A6 has emerged as a versatile Ca^2+^/Zn^2+^ sensor central to nervous system physiology and pathology. Its regulated expression underpins key roles in development and homeostasis. By engaging a broad, calcium-dependent network of cytoskeletal, chaperone, nuclear, and signaling proteins, S100A6 functions as a pivotal hub that converts ionic signals into coordinated cellular responses. Across neurological diseases, S100A6 acts as a dynamically regulated active participant. In disorders like AD and ALS, it connects key pathological features such as protein aggregation, metal dyshomeostasis, and neuroinflammation. In oncology, its roles are context-specific, functioning as an oncogenic driver in glioblastoma, an epigenetically silenced marker in medulloblastoma, and a diagnostic aid in peripheral nerve tumors. It is further implicated in the pathophysiology of epilepsy, TBI, and autoimmune encephalitis. This functional duality and context-dependence highlight the need for mechanistic, disease-specific understanding. Future research aimed at deciphering its precise spatiotemporal roles is essential. Success will pave the way for developing S100A6-based diagnostics and targeted therapies, offering new avenues to address challenging neurological diseases.

## References

[B1] Anfinogenova N. D. Quinn M. T. Schepetkin I. A. Atochin D. N. (2020). Alarmins and c-Jun N-Terminal kinase (JNK) signaling in neuroinflammation. Cells 9 (11), 2350. 10.3390/cells9112350 33114371 PMC7693759

[B2] Astillero-Lopez V. Gonzalez-Rodriguez M. Villar-Conde S. Flores-Cuadrado A. Martinez-Marcos A. Ubeda-Banon I. (2022). Neurodegeneration and astrogliosis in the entorhinal cortex in Alzheimer’s disease: stereological layer-specific assessment and proteomic analysis. Alzheimers Dement. 18 (12), 2468–2480. 10.1002/alz.12580 35142030

[B3] Bartkowska K. Swiatek I. Aniszewska A. Jurewicz E. Turlejski K. Filipek A. (2017). Stress-dependent changes in the CacyBP/SIP interacting protein S100A6 in the mouse brain. PLoS One 12 (1), e0169760. 10.1371/journal.pone.0169760 28068373 PMC5221789

[B4] Basso M. Bonetto V. (2016). Extracellular vesicles and a novel form of communication in the brain. Front. Neurosci. 10, 127. 10.3389/fnins.2016.00127 27065789 PMC4814526

[B5] Boom A. Pochet R. Authelet M. Pradier L. Borghgraef P. Van Leuven F. (2004). Astrocytic calcium/zinc binding protein S100A6 over expression in Alzheimer’s disease and in PS1/APP transgenic mice models. Biochim. Biophys. Acta 1742 (1-3), 161–168. 10.1016/j.bbamcr.2004.09.011 15590066

[B6] Botelho H. M. Leal S. S. Cardoso I. Yanamandra K. Morozova-Roche L. A. Fritz G. (2012). S100A6 amyloid fibril formation is calcium-modulated and enhances superoxide dismutase-1 (SOD1) aggregation. J. Biol. Chem. 287 (50), 42233–42242. 10.1074/jbc.M112.396416 23076148 PMC3516767

[B7] Cacheaux L. P. Ivens S. David Y. Lakhter A. J. Bar-Klein G. Shapira M. (2009). Transcriptome profiling reveals TGF-beta signaling involvement in epileptogenesis. J. Neurosci. 29 (28), 8927–8935. 10.1523/JNEUROSCI.0430-09.2009 19605630 PMC2875073

[B8] Calabretta B. Battini R. Kaczmarek L. de Riel J. K. Baserga R. (1986). Molecular cloning of the cDNA for a growth factor-inducible gene with strong homology to S-100, a calcium-binding protein. J. Biol. Chem. 261 (27), 12628–12632. 10.1016/S0021-9258(18)67137-6 3755724

[B9] Camby I. Nagy N. Lopes M. B. Schäfer B. W. Maurage C. A. Ruchoux M. M. (1999). Supratentorial pilocytic astrocytomas, astrocytomas, anaplastic astrocytomas and glioblastomas are characterized by a differential expression of S100 proteins. Brain Pathol. 9 (1), 1–19. 10.1111/j.1750-3639.1999.tb00205.x 9989446 PMC8098381

[B10] Camby I. Lefranc F. Titeca G. Neuci S. Fastrez M. Dedecken L. (2000). Differential expression of S100 calcium-binding proteins characterizes distinct clinical entities in both WHO grade II and III astrocytic tumours. Neuropathol. Appl. Neurobiol. 26 (1), 76–90. 10.1046/j.1365-2990.2000.00223.x 10736069

[B11] Chauhan S. Behl T. Sehgal A. Singh S. Sharma N. Gupta S. (2022). Understanding the intricate role of exosomes in pathogenesis of Alzheimer's disease. Neurotox. Res. 40 (6), 1758–1773. 10.1007/s12640-022-00621-4 36564606

[B12] Chen T. Ruan Y. Ji L. Cai J. Tong M. Xue Y. (2024). S100A6 drives lymphatic metastasis of liver cancer via activation of the RAGE/NF-kB/VEGF-D pathway. Cancer Lett. 587, 216709. 10.1016/j.canlet.2024.216709 38350547

[B13] Cristóvão J. S. Gomes C. M. (2019). S100 proteins in alzheimer’s disease. Front. Neurosci. 13, 463. 10.3389/fnins.2019.00463 31156365 PMC6532343

[B14] De Bastiani M. A. Bellaver B. Carello-Collar G. Zimmermann M. Kunach P. Lima-Filho R. A. S. (2023). Cross-species comparative hippocampal transcriptomics in Alzheimer’s disease. iScience 27 (1), 108671. 10.1016/j.isci.2023.108671 38292167 PMC10824791

[B15] Ding H. Yu J. Chang W. Liu F. He Z. (2020). Searching for differentially expressed proteins in spinal cord injury based on the proteomics analysis. Life Sci. 242, 117235. 10.1016/j.lfs.2019.117235 31887301

[B16] Doi S. Fujioka N. Ohtsuka S. Kondo R. Yamamoto M. Denda M. (2021). Regulation of the tubulin polymerization-promoting protein by Ca2+/S100 proteins. Cell Calcium 96, 102404. 10.1016/j.ceca.2021.102404 33831707

[B17] Donato R. Sorci G. Giambanco I. (2017). S100A6 protein: functional roles. Cell Mol. Life Sci. 74 (15), 2749–2760. 10.1007/s00018-017-2526-9 28417162 PMC11107720

[B18] Fang B. Liang M. Yang G. Ye Y. Xu H. He X. (2014). Expression of S100A6 in rat hippocampus after traumatic brain injury due to lateral head acceleration. Int. J. Mol. Sci. 15 (4), 6378–6390. 10.3390/ijms15046378 24739809 PMC4013634

[B19] Filipek A. Leśniak W. (2020). S100A6 and its brain ligands in neurodegenerative disorders. Int. J. Mol. Sci. 21 (11), 3979. 10.3390/ijms21113979 32492924 PMC7313082

[B20] Filipek A. Wojda U. (1996). p30, a novel protein target of mouse calcyclin (S100A6). Biochem. J. 320 (Pt 2), 585–587. 10.1042/bj3200585 8973570 PMC1217969

[B21] Filipek A. Heizmann C. W. Kuźnicki J. (1990). Calcyclin is a calcium and zinc binding protein. FEBS Lett. 264 (2), 263–266. 10.1016/0014-5793(90)80263-i 2358072

[B22] Filipek A. Zasada A. Wojda U. Makuch R. Dabrowska R. (1996). Characterization of chicken gizzard calcyclin and examination of its interaction with caldesmon. Comp. Biochem. Physiol. B Biochem. Mol. Biol. 113 (4), 745–752. 10.1016/0305-0491(95)02095-0 8925441

[B23] Filipek A. Jastrzebska B. Nowotny M. Kwiatkowska K. Hetman M. Surmacz L. (2002). Ca2+-dependent translocation of the calcyclin-binding protein in neurons and neuroblastoma NB-2a cells. J. Biol. Chem. 277 (23), 21103–21109. 10.1074/jbc.M111010200 11927578

[B24] Filipek A. Schneider G. Mietelska A. Figiel I. Niewiadomska G. (2008). Age-dependent changes in neuronal distribution of CacyBP/SIP: comparison to tubulin and the tau protein. J. Neural Transm. (Vienna) 115 (9), 1257–1264. 10.1007/s00702-008-0062-3 18506390

[B25] Fullen D. R. Lowe L. Su L. D. (2003). Antibody to S100a6 protein is a sensitive immunohistochemical marker for neurothekeoma. J. Cutan. Pathol. 30 (2), 118–122. 10.1034/j.1600-0560.2002.00032.x 12641790

[B26] Golitsina N. L. Kordowska J. Wang C. L. Lehrer S. S. (1996). Ca2+-dependent binding of calcyclin to muscle tropomyosin. Biochem. Biophys. Res. Commun. 220 (2), 360–365. 10.1006/bbrc.1996.0410 8645310

[B27] Hagmeyer S. Romão M. A. Cristóvão J. S. Vilella A. Zoli M. Gomes C. M. (2019). Distribution and relative abundance of S100 proteins in the brain of the APP23 Alzheimer's disease model mice. Front. Neurosci. 13, 640. 10.3389/fnins.2019.00640 31281238 PMC6596341

[B28] Haldar B. Hamilton C. L. Solodushko V. Abney K. A. Alexeyev M. Honkanen R. E. (2020). S100A6 is a positive regulator of PPP5C-FKBP51-dependent regulation of endothelial calcium signaling. FASEB J. 34 (2), 3179–3196. 10.1096/fj.201901777R 31916625 PMC7401177

[B29] Han R. Chi G. Sun D. Xu Z. Zhou L. Xu K. (2025). Single-cell and spatial transcriptomics reveal lactylation-associated tumor cell clusters and define a prognostic risk model in glioblastoma. BMC Cancer 25, 1901. 10.1186/s12885-025-15291-6 41275140 PMC12750639

[B30] Hong B. Zhang H. Xiao Y. Shen L. Qian Y. (2023). S100A6 is a potential diagnostic and prognostic biomarker for human glioma. Oncol. Lett. 26 (4), 458. 10.3892/ol.2023.14045 37736555 PMC10509776

[B31] Hoyaux D. Alao J. Fuchs J. Kiss R. Keller B. Heizmann C. W. (2000). S100A6, a calcium- and zinc-binding protein, is overexpressed in SOD1 mutant mice, a model for amyotrophic lateral sclerosis. Biochim. Biophys. Acta 1498 (2-3), 264–272. 10.1016/s0167-4889(00)00101-4 11108968

[B32] Hoyaux D. Boom A. Van den Bosch L. Belot N. Martin J. J. Heizmann C. W. (2002). S100A6 overexpression within astrocytes associated with impaired axons from both ALS mouse model and human patients. J. Neuropathol. Exp. Neurol. 61 (8), 736–744. 10.1093/jnen/61.8.736 12152788

[B33] Iridoy M. O. Zubiri I. Zelaya M. V. Martinez L. Ausín K. Lachen-Montes M. (2018). Neuroanatomical quantitative proteomics reveals common pathogenic biological routes between Amyotrophic lateral sclerosis (ALS) and frontotemporal dementia (FTD). Int. J. Mol. Sci. 20 (1), 4. 10.3390/ijms20010004 30577465 PMC6337647

[B34] Jastrzebska B. Filipek A. Nowicka D. Kaczmarek L. Kúznicki J. (2000). Calcyclin (S100A6) binding protein (CacyBP) is highly expressed in brain neurons. J. Histochem Cytochem 48 (9), 1195–1202. 10.1177/002215540004800903 10950876

[B35] Jurewicz E. Bednarczyk J. Bot A. Lukasiuk K. Filipek A. (2013). Status epilepticus induces long lasting increase in S100A6 expression in astrocytes. Neurochem. Res. 38 (9), 1941–1948. 10.1007/s11064-013-1100-6 23817846

[B36] Jurewicz E. Wyroba E. Filipek A. (2018). Tubulin-dependent secretion of S100A6 and cellular signaling pathways activated by S100A6-integrin β1 interaction. Cell Signal 42, 21–29. 10.1016/j.cellsig.2017.10.004 29020611

[B37] Jurewicz E. Robaszkiewicz K. Moraczewska J. Filipek A. (2020). Binding of S100A6 to actin and the actin-tropomyosin complex. Sci. Rep. 10 (1), 12824. 10.1038/s41598-020-69752-y 32733033 PMC7393103

[B38] Khan M. I. Dowarha D. Katte R. Chou R. H. Filipek A. Yu C. (2019). Lysozyme as the anti-proliferative agent to block the interaction between S100A6 and the RAGE V domain. PLoS One 14 (5), e0216427. 10.1371/journal.pone.0216427 31071146 PMC6508705

[B39] Kilanczyk E. Filipek S. Jastrzebska B. Filipek A. (2009). CacyBP/SIP binds ERK1/2 and affects transcriptional activity of Elk-1. Biochem. Biophys. Res. Commun. 380 (1), 54–59. 10.1016/j.bbrc.2009.01.026 19166809

[B40] Kilańczyk E. Graczyk A. Ostrowska H. Kasacka I. Leśniak W. Filipek A. (2012). S100A6 is transcriptionally regulated by β-catenin and interacts with a novel target, lamin A/C, in colorectal cancer cells. Cell Calcium 51 (6), 470–477. 10.1016/j.ceca.2012.04.005 22560296

[B41] Kitagawa K. Skowyra D. Elledge S. J. Harper J. W. Hieter P. (1999). SGT1 encodes an essential component of the yeast kinetochore assembly pathway and a novel subunit of the SCF ubiquitin ligase complex. Mol. Cell 4 (1), 21–33. 10.1016/s1097-2765(00)80184-7 10445024

[B42] Kjell J. Fischer-Sternjak J. Thompson A. J. Friess C. Sticco M. J. Salinas F. (2020). Defining the adult neural stem cell niche proteome identifies key regulators of adult neurogenesis. Cell Stem Cell 26 (2), 277–293.e8. 10.1016/j.stem.2020.01.002 32032526 PMC7005820

[B43] Kobori N. Clifton G. L. Dash P. (2002). Altered expression of novel genes in the cerebral cortex following experimental brain injury. Brain Res. Mol. Brain Res. 104 (2), 148–158. 10.1016/s0169-328x(02)00331-5 12225869

[B44] Króliczak W. Pietrzak M. Puzianowska-Kuznicka M. (2008). P53-dependent suppression of the human calcyclin gene (S100A6): the role of Sp1 and of NFkappaB. Acta Biochim. Pol. 55 (3), 559–570. 10.18388/abp.2008_3062 18714402

[B45] Kucharczak J. Pannequin J. Camby I. Decaestecker C. Kiss R. Martinez J. (2001). Gastrin induces over-expression of genes involved in human U373 glioblastoma cell migration. Oncogene 20 (48), 7021–7028. 10.1038/sj.onc.1204882 11704826

[B46] Leclerc E. Fritz G. Weibel M. Heizmann C. W. Galichet A. (2007). S100B and S100A6 differentially modulate cell survival by interacting with distinct RAGE (receptor for advanced glycation end products) immunoglobulin domains. J. Biol. Chem. 282 (43), 31317–31331. 10.1074/jbc.M703951200 17726019

[B47] Lee T. S. Mane S. Eid T. Zhao H. Lin A. Guan Z. (2007). Gene expression in temporal lobe epilepsy is consistent with increased release of glutamate by astrocytes. Mol. Med. 13 (1-2), 1–13. 10.2119/2006-00079.Lee 17515952 PMC1869627

[B48] Leśniak W. Filipek A. (2023). S100A6 protein-expression and function in norm and pathology. Int. J. Mol. Sci. 24 (2), 1341. 10.3390/ijms24021341 36674873 PMC9866648

[B49] Li Y. Chen L. Li Z. Song Y. Yuan Y. Liu T. (2021). Potential serum biomarkers for postoperative neurocognitive disorders based on proteomic analysis of cognitive-related brain regions. Front. Aging Neurosci. 13, 741263. 10.3389/fnagi.2021.741263 34658843 PMC8511679

[B50] Lin C. H. Li S. C. Lin M. H. Ho C. J. Lu Y. T. Lin Y. (2023). S100A6 participates in initiation of autoimmune encephalitis and is under epigenetic control. Brain Behav. 13 (3), e2897. 10.1002/brb3.2897 36748983 PMC10013942

[B51] Lindsey J. C. Lusher M. E. Anderton J. A. Gilbertson R. J. Ellison D. W. Clifford S. C. (2007). Epigenetic deregulation of multiple S100 gene family members by differential hypomethylation and hypermethylation events in medulloblastoma. Br. J. Cancer 97 (2), 267–274. 10.1038/sj.bjc.6603852 17579622 PMC2360310

[B52] Marenholz I. Heizmann C. W. Fritz G. (2004). S100 proteins in mouse and man: from evolution to function and pathology (including an update of the nomenclature). Biochem. Biophys. Res. Commun. 322 (4), 1111–1122. 10.1016/j.bbrc.2004.07.096 15336958

[B53] Matsuzawa S. I. Reed J. C. (2001). Siah-1, SIP, and Ebi collaborate in a novel pathway for beta-catenin degradation linked to p53 responses. Mol. Cell 7 (5), 915–926. 10.1016/s1097-2765(01)00242-8 11389839

[B54] Michetti F. Clementi M. E. Di Liddo R. Valeriani F. Ria F. Rende M. (2023). The S100B protein: a multifaceted pathogenic factor more than a biomarker. Int. J. Mol. Sci. 24 (11), 9605. 10.3390/ijms24119605 37298554 PMC10253509

[B55] Misago N. Satoh T. Narisawa Y. (2004). Cellular neurothekeoma with histiocytic differentiation. J. Cutan. Pathol. 31 (8), 568–572. 10.1111/j.0303-6987.2004.00223.x 15268715

[B56] Nowotny M. Spiechowicz M. Jastrzebska B. Filipek A. Kitagawa K. Kuznicki J. (2003). Calcium-regulated interaction of Sgt1 with S100A6 (calcyclin) and other S100 proteins. J. Biol. Chem. 278 (29), 26923–26928. 10.1074/jbc.M211518200 12746458

[B57] Otterbein L. R. Kordowska J. Witte-Hoffmann C. Wang C. L. Dominguez R. (2002). Crystal structures of S100A6 in the Ca(2+)-free and Ca(2+)-bound states: the calcium sensor mechanism of S100 proteins revealed at atomic resolution. Structure 10 (4), 557–567. 10.1016/s0969-2126(02)00740-2 11937060

[B58] Plaza J. A. Torres-Cabala C. Evans H. Diwan A. H. Prieto V. G. (2009). Immunohistochemical expression of S100A6 in cellular neurothekeoma: clinicopathologic and immunohistochemical analysis of 31 cases. Am. J. Dermatopathol. 31 (5), 419–422. 10.1097/DAD.0b013e3181a13afc 19542912

[B59] Prus W. Filipek A. (2011). S100A6 mediates nuclear translocation of Sgt1: a heat shock-regulated protein. Amino Acids 41 (4), 781–787. 10.1007/s00726-010-0526-2 20213445

[B60] Qureshi I. A. Mehler M. F. (2018). Epigenetic mechanisms underlying nervous system diseases. Handb. Clin. Neurol. 147, 43–58. 10.1016/B978-0-444-63233-3.00005-1 29325627 PMC6822391

[B61] Rehman I. Cross S. S. Catto J. W. Leiblich A. Mukherjee A. Azzouzi A. R. (2005). Promoter hyper-methylation of calcium binding proteins S100A6 and S100A2 in human prostate cancer. Prostate 65 (4), 322–330. 10.1002/pros.20302 16015609

[B62] Rezvanpour A. Shaw G. S. (2009). Unique S100 target protein interactions. Gen. Physiol. Biophys. 28, F39–F46. 20093725

[B63] Robaszkiewicz K. Jurewicz E. Moraczewska J. Filipek A. (2021). Ca2+-dependent binding of S100A6 to cofilin-1 regulates actin filament polymerization-depolymerization dynamics. Cell Calcium 99, 102457. 10.1016/j.ceca.2021.102457 34464867

[B64] Rossi S. Milani M. Della Valle I. Apolloni S. (2025). Transcriptomic profiling of symptomatic and end-stage SOD1-G93A transgenic mice reveals extracellular matrix components as key players in ALS pathogenesis. Biochim. Biophys. Acta Mol. Basis Dis. 1871 (4), 167707. 10.1016/j.bbadis.2025.167707 39922547

[B65] Sakane K. Nishiguchi M. Denda M. Yamagchi F. Magari M. Kanayama N. (2017). Identification and characterization of a centrosomal protein, FOR20 as a novel S100A6 target. Biochem. Biophys. Res. Commun. 491 (4), 980–985. 10.1016/j.bbrc.2017.07.161 28765046

[B66] Sastry M. Ketchem R. R. Crescenzi O. Weber C. Lubienski M. J. Hidaka H. (1998). The three-dimensional structure of Ca(2+)-bound calcyclin: implications for Ca(2+)-signal transduction by S100 proteins. Structure 6 (2), 223–231. 10.1016/s0969-2126(98)00023-9 9519412

[B67] Saunders A. Macosko E. Z. Wysoker A. Goldman M. Krienen F. M. de Rivera H. (2018). Molecular diversity and specializations among the cells of the adult mouse brain. Cell 174 (4), 1015–1030.e16. 10.1016/j.cell.2018.07.028 30096299 PMC6447408

[B68] Shimamoto S. Takata M. Tokuda M. Oohira F. Tokumitsu H. Kobayashi R. (2008). Interactions of S100A2 and S100A6 with the tetratricopeptide repeat proteins, Hsp90/Hsp70-organizing protein and kinesin light chain. J. Biol. Chem. 283 (42), 28246–28258. 10.1074/jbc.M801473200 18669640 PMC2661394

[B69] Shimamoto S. Kubota Y. Tokumitsu H. Kobayashi R. (2010). S100 proteins regulate the interaction of Hsp90 with Cyclophilin 40 and FKBP52 through their tetratricopeptide repeats. FEBS Lett. 584 (6), 1119–1125. 10.1016/j.febslet.2010.02.055 20188096

[B70] Shimamoto S. Kubota Y. Yamaguchi F. Tokumitsu H. Kobayashi R. (2013). Ca2+/S100 proteins act as upstream regulators of the chaperone-associated ubiquitin ligase CHIP (C terminus of Hsc70-interacting protein). J. Biol. Chem. 288 (10), 7158–7168. 10.1074/jbc.M112.436758 23344957 PMC3591625

[B71] Shimamoto S. Tsuchiya M. Yamaguchi F. Kubota Y. Tokumitsu H. Kobayashi R. (2014). Ca2+/S100 proteins inhibit the interaction of FKBP38 with Bcl-2 and Hsp90. Biochem. J. 458 (1), 141–152. 10.1042/BJ20130924 24295050

[B72] Simon M. A. Ecsédi P. Kovács G. M. Póti Á. L. Reményi A. Kardos J. (2020). High-throughput competitive fluorescence polarization assay reveals functional redundancy in the S100 protein family. FEBS J. 287 (13), 2834–2846. 10.1111/febs.15175 31837246

[B73] Słomnicki L. P. Leśniak W. (2010). S100A6 (calcyclin) deficiency induces senescence-like changes in cell cycle, morphology and functional characteristics of mouse NIH 3T3 fibroblasts. J. Cell Biochem. 109 (3), 576–584. 10.1002/jcb.22434 20013795

[B74] Słomnicki Ł. P. Nawrot B. Leśniak W. (2009). S100A6 binds p53 and affects its activity. Int. J. Biochem. Cell Biol. 41 (4), 784–790. 10.1016/j.biocel.2008.08.007 18765292

[B75] Spiechowicz M. Filipek A. (2005). The expression and function of Sgt1 protein in eukaryotic cells. Acta Neurobiol. Exp. (Wars). 65 (2), 161–165. 10.55782/ane-2005-1549 15960300

[B76] Stuart T. Satija R. (2019). Integrative single-cell analysis. Nat. Rev. Genet. 20 (5), 257–272. 10.1038/s41576-019-0093-7 30696980

[B77] Takata M. Shimamoto S. Yamaguchi F. Tokuda M. Tokumitsu H. Kobayashi R. (2010). Regulation of nuclear localization signal-importin α interaction by Ca2+/S100A6. FEBS Lett. 584 (22), 4517–4523. 10.1016/j.febslet.2010.09.052 20965181

[B78] Tang Y. Qing C. Wang J. Zeng Z. (2020). DNA methylation-based diagnostic and prognostic biomarkers for glioblastoma. Cell Transpl. 29, 963689720933241. 10.1177/0963689720933241 32510239 PMC7563836

[B79] Teunissen C. E. Verberk I. M. W. Thijssen E. H. Vermunt L. Hansson O. Zetterberg H. (2022). Blood-based biomarkers for Alzheimer's disease: towards clinical implementation. Lancet Neurol. 21 (1), 66–77. 10.1016/s1474-4422(21)00361-6 34838239

[B80] Tian Z. Y. Wang C. Y. Wang T. Li Y. C. Wang Z. Y. (2019). Glial S100A6 degrades β-amyloid aggregation through targeting competition with zinc ions. Aging Dis. 10 (4), 756–769. 10.14336/AD.2018.0912 31440382 PMC6675528

[B81] Tiu S. C. Chan W. Y. Heizmann C. W. Schäfer B. W. Shu S. Y. Yew D. T. (2000). Differential expression of S100B and S100A6(1) in the human fetal and aged cerebral cortex. Brain Res. Dev. Brain Res. 119 (2), 159–168. 10.1016/s0165-3806(99)00151-0 10675765

[B82] Tsai M. H. Lin C. H. Tsai K. W. Lin M. H. Ho C. J. Lu Y. T. (2019). S100A6 promotes B lymphocyte penetration through the blood-brain barrier in Autoimmune encephalitis. Front. Genet. 10, 1188. 10.3389/fgene.2019.01188 31850060 PMC6901080

[B83] van Dieck J. Brandt T. Teufel D. P. Veprintsev D. B. Joerger A. C. Fersht A. R. (2010). Molecular basis of S100 proteins interacting with the p53 homologs p63 and p73. Oncogene 29 (14), 2024–2035. 10.1038/onc.2009.490 20140014

[B84] Wang X. H. Du H. Li L. Shao D. F. Zhong X. Y. Hu Y. (2017). Increased expression of S100A6 promotes cell proliferation in gastric cancer cells. Oncol. Lett. 13 (1), 222–230. 10.3892/ol.2016.5419 28123545 PMC5245149

[B85] Wang T. Han S. Du G. (2021a). S100A6 represses Calu-6 lung cancer cells growth *via* inhibiting cell proliferation, migration, invasion and enhancing apoptosis. Cell Biochem. Funct. 39 (6), 771–779. 10.1002/cbf.3639 34008212 PMC8453982

[B86] Wang T. Du G. Wang D. (2021b). The S100 protein family in lung cancer. Clin. Chim. Acta 520, 67–70. 10.1016/j.cca.2021.05.028 34089725

[B87] Wang Y. Kang X. Kang X. Yang F. (2023). S100A6: molecular function and biomarker role. Biomark. Res. 11 (1), 78. 10.1186/s40364-023-00515-3 37670392 PMC10481514

[B88] Wasik U. Schneider G. Mietelska-Porowska A. Mazurkiewicz M. Fabczak H. Weis S. (2013). Calcyclin binding protein and Siah-1 interacting protein in Alzheimer’s disease pathology: neuronal localization and possible function. Neurobiol. Aging 34 (5), 1380–1388. 10.1016/j.neurobiolaging.2012.11.007 23260124

[B89] Wasik U. Kadziolka B. Kilanczyk E. Filipek A. (2016). Influence of S100A6 on CacyBP/SIP phosphorylation and Elk-1 transcriptional activity in neuroblastoma NB2a cells. J. Cell Biochem. 117 (1), 126–131. 10.1002/jcb.25257 26085436

[B90] Wiesenfarth M. Dorst J. Brenner D. Elmas Z. Parlak Ö. Uzelac Z. (2024). Effects of tofersen treatment in patients with SOD1-ALS in a “real-world” setting - a 12-month multicenter cohort study from the German early access program. EClinicalMedicine 69, 102495. 10.1016/j.eclinm.2024.102495 38384337 PMC10878861

[B91] Wills F. L. McCubbin W. D. Kay C. M. (1994). Smooth muscle calponin-caltropin interaction: effect on biological activity and stability of calponin. Biochemistry 33 (18), 5562–5569. 10.1021/bi00184a027 8180179

[B92] Wruck W. Schröter F. Adjaye J. (2016). Meta-Analysis of transcriptome data related to hippocampus biopsies and iPSC-Derived neuronal cells from alzheimer’s disease patients reveals an Association with FOXA1 and FOXA2 gene regulatory networks. J. Alzheimers Dis. 50 (4), 1065–1082. 10.3233/JAD-150733 26890743

[B93] Yamada J. Jinno S. (2012). Upregulation of calcium binding protein, S100A6, in activated astrocytes is linked to glutamate toxicity. Neuroscience 226, 119–129. 10.1016/j.neuroscience.2012.08.068 22982625

[B94] Yamada J. Jinno S. (2014). S100A6 (calcyclin) is a novel marker of neural stem cells and astrocyte precursors in the subgranular zone of the adult mouse hippocampus. Hippocampus 24 (1), 89–101. 10.1002/hipo.22207 24115312

[B95] Yamaguchi F. Umeda Y. Shimamoto S. Tsuchiya M. Tokumitsu H. Tokuda M. (2012). S100 proteins modulate protein phosphatase 5 function: a link between CA2+ signal transduction and protein dephosphorylation. J. Biol. Chem. 287 (17), 13787–13798. 10.1074/jbc.M111.329771 22399290 PMC3340197

[B96] Yamaguchi F. Yamamura S. Shimamoto S. Tokumitsu H. Tokuda M. Kobayashi R. (2014). Suramin is a novel activator of PP5 and biphasically modulates S100-activated PP5 activity. Appl. Biochem. Biotechnol. 172 (1), 237–247. 10.1007/s12010-013-0522-6 24068474

[B97] Yamashita N. Kosaka K. Ilg E. C. Schäfer B. W. Heizmann C. W. Kosaka T. (1997). Selective association of S100A6 (calcyclin)-immunoreactive astrocytes with the tangential migration pathway of subventricular zone cells in the rat. Brain Res. 778 (2), 388–392. 10.1016/s0006-8993(97)01025-1 9459556

[B98] Yamashita N. Ilg E. C. Schäfer B. W. Heizmann C. W. Kosaka T. (1999). Distribution of a specific calcium-binding protein of the S100 protein family, S100A6 (calcyclin), in subpopulations of neurons and glial cells of the adult rat nervous system. J. Comp. Neurol. 404 (2), 235–257. 10.1002/(SICI)1096-9861(19990208)404:2<235::AID-CNE8>3.0.CO;2-7 9934997

[B99] Yang F. Ma J. Zhu D. Wang Z. Li Y. He X. (2023). The role of S100A6 in human diseases: molecular mechanisms and therapeutic potential. Biomolecules 13 (7), 1139. 10.3390/biom13071139 37509175 PMC10377078

[B100] Yang Y. Hong Y. Zhao K. Huang M. Li W. Zhang K. (2024). Spatial transcriptomics analysis identifies therapeutic targets in diffuse high-grade gliomas. Front. Mol. Neurosci. 17, 1466302. 10.3389/fnmol.2024.1466302 39530009 PMC11552449

[B101] Yatime L. Betzer C. Jensen R. K. Mortensen S. Jensen P. H. Andersen G. R. (2016). The structure of the RAGE:S100A6 complex reveals a unique mode of homodimerization for S100 proteins. Structure 24 (12), 2043–2052. 10.1016/j.str.2016.09.011 27818100

[B102] Zhang R. Lu J. Kong X. Jin L. Luo C. (2013). Targeting epigenetics in nervous system disease. CNS Neurol. Disord. Drug Targets 12 (1), 126–141. 10.2174/1871527311312010018 23244434

[B103] Zhang Y. Yang X. Zhu X. L. Bai H. Wang Z. Z. Zhang J. J. (2021). S100A gene family: immune-related prognostic biomarkers and therapeutic targets for low-grade glioma. Aging (Albany NY) 13 (11), 15459–15478. 10.18632/aging.203103 34148033 PMC8221329

