# Supplementary material for: Multifaceted roles of S100A6 in neurological disorders: expression, interaction networks, and clinical implications
Source: Front Pharmacol. 2026 Aug 12;17:1890559. doi: 10.3389/fphar.2026.1890559 (PMC13507526; doi:10.3389/fphar.2026.1890559)
Supplement: Supplementary file 1 [file DataSheet1.doc]

**Supplementary Materials**

 **Supplementary Figure 1. Schematic representation of intracellular networks mediated by S100A6.** The diagram depicts the major intracellular protein targets of S100A6, categorized by their functional roles. S100A6 engages: (i) signaling regulators (CacyBP/SIP, Sgt1) that modulate microtubule dynamics, cell growth, differentiation, and also control cell cycle regulation and chaperone complex assembly; (ii) TPR domain-containing chaperones and co-chaperones (Hop, Cyp40, FKBP52, CHIP, PP5) involved in protein folding/degradation, intracellular transport, mitochondrial function, and phospho-signaling; and (iii) cytoskeletal proteins (actin, tropomyosin, actin-binding proteins, α/β-tubulin heterodimers, TPPP) that regulate microfilament and microtubule dynamics, as well as centrosome and cilia function.

**Supplementary Figure 2. Extracellular roles of S100A6.** Secreted S100A6 functions as a damage-associated molecular pattern (DAMP) through Ca^2+^-dependent binding to two major cell surface receptors. Engagement with the receptor of advanced glycation end-products (RAGE) triggers pro-inflammatory reactive oxygen species (ROS)/c-Jun N-terminal kinase (JNK) signaling, whereas interaction with integrin β1 activates integrin-linked kinase (ILK)/focal adhesion kinase (FAK)/p21-activated kinase (PAK) cascades. Collectively, these pathways modulate cell proliferation, apoptosis, adhesion, and motility.

**Supplementary Figure 3. Nuclear interaction network of S100A6.** S100A6 regulates nuclear processes through interactions with three functional classes: a) Nuclear envelope organization - it binds lamin A/C to maintain nuclear integrity; b) Nucleocytoplasmic transport - it interacts with importin α to inhibit nuclear localization signals (NLS)-mediated protein import; c) transcriptional regulation - it binds p53 family members to modulate cell cycle arrest and apoptosis.
